# A pre/post study of a narrative “IDEAS” intervention’s impact on provider stigma and feasibility of collecting transgender and gender diverse veteran care experiences

**DOI:** 10.1186/s12889-025-24494-2

**Published:** 2025-10-01

**Authors:** Sally Wasmuth, Johnna Belkiewitz, Heather A. Sperry, Francis Pike, Mio Ueki, Dawn M. Bravata

**Affiliations:** 1 School of Health and Human Sciences Occupational Therapy Program, Indiana University Indianapolis, 1050 Wishard Boulevard, Indianapolis, IN 46202 USA; 2 Center for Health Information and Communication, Roudebush VA Medical Center, 1481 W. 10th Street, Indianapolis, IN 46202 USA; 3 Indiana University Indianapolis School of Medicine, 340 W. 10th Street, Indianapolis, IN 46202 USA

**Keywords:** Implementation research, Health services, Theatre

## Abstract

**Background:**

Transgender and gender diverse (TGD) Veterans face significant health disparities, including greater psychiatric comorbidities and suicide attempts than the general Veteran population, which are often exacerbated by provider stigma. Innovations targeting provider stigma are warranted to address this healthcare concern. IDEAS is a film-based intervention for reducing provider stigma to improve healthcare for TGD Veterans.

**Methods:**

We used a hybrid type 2 design to examine IDEAS implementation acceptability, feasibility, and effectiveness (decreased provider stigma). We also tested the feasibility of a nested data collection methodology that would allow future studies to assess whether IDEAS implementation with providers impacted care from TGD patients’ perspectives. The study involved primary care and mental health providers from a Midwestern VA Medical Center and TGD Veterans with appointments within six months before and after IDEAS. Providers completed the Acceptability of Intervention Measure (AIM) and Feasibility of Intervention Measure (FIM) post-IDEAS, and the Acceptance and Action Questionnaire – Stigma (AAQ-S) pre and post-intervention. TGD Veterans completed the Consumer Assessment of Healthcare Providers and Systems Cultural Competence Item Set (CAHPS-CC) and an author-created survey pre and post IDEAS. AIM and FIM scores were averaged across providers and paired two samples t tests were used to determine change in provider stigma. TGD Veteran recruitment and response rates were tracked, and pre/post survey scores were averaged to illustrate trends in pre/post score differences.

**Results:**

Average AIM and FIM scores were above 4 on a scale of 1 (‘highly disagree’) to 5 (‘highly agree’), suggesting providers ‘agree’ that the IDEAS intervention was acceptable and feasible. Provider stigma measured by the AAQ-S decreased significantly post-intervention (*p* < 0.01). Feasibility benchmarks for testing a nested data collection design to obtain information on TGD Veteran healthcare experiences pre/post IDEAS were met. Preliminary exploration of the sum/difference of average pre versus posttest item scores of Veteran data suggested improved perceptions of provider communication, trust.

**Conclusions:**

IDEAS shows promise in reducing provider stigma and enhancing experiences of TGD Veterans. Further research is needed to explore the long-term impact of such interventions on TGD Veteran health outcomes and to refine assessment tools for capturing nuanced patient experiences.

**Supplementary Information:**

The online version contains supplementary material available at 10.1186/s12889-025-24494-2.

## Introduction

Nearly 20% of the adult transgender population has served in the United States Military [1]. Development and implementation of evidence-based, stigma-reduction interventions are urgent and critical to support the health and safety of transgender and gender-diverse (TGD) Veterans [2]. In a study examining medical records of over 32,000 Veterans, TGD Veterans were twice as likely to perform suicide compared to cisgender Veterans [3]. A case-control study of over 5,000 transgender Veterans receiving VA healthcare found “statistically significant disparities were present in the [transgender] cohort for all 10 mental health conditions examined, including depression, suicidality, serious mental illnesses, and post-traumatic stress disorder.” [4] The authors further report that transgender Veterans were “more likely to have been homeless [and] to have reported sexual trauma while on active duty.” [4] Disaffirming healthcare experiences deter TGD Veterans from seeking needed medical services and thereby drive health disparities [3]. A quality improvement project using a narrative medicine approach illuminated that stigma exacerbates health disparities by dissuading TGD Veterans from revealing their identities to healthcare providers who may not ask about gender identity, limiting delivery of affirming, comprehensive care [5]. By contrast, affirming policies can decrease harm – for instance, a study of 1640 Veterans found living in a state with employment non-discrimination protections for TGD Veterans was associated with a 26% decreased likelihood of mood disorders and 43% decreased likelihood of self-harm [6]. VA research has examined health services utilization and barriers and facilitators to care from the perspectives of TGD Veterans and VA healthcare providers, but implementation of Veteran-informed, evidence-based interventions to improve affirming TGD patient care is needed particularly for providers who may not otherwise seek out this type of training [7, 8].

‘Identity Development Evolution and Sharing’ (IDEAS) is an evidence-supported methodology developed by occupational therapists to reduce provider stigma across healthcare disciplines [9]. Occupational therapists are committed to promoting justice, especially among people who experience healthcare disparities. In this vein, occupational therapist researchers created the IDEAS method to promote justice for the people harmed by provider stigma. A detailed explanation of the development of the IDEAS methodology can be found in prior work [10]. IDEAS interventions have been developed to address provider stigma across disciplines toward several populations including Black women, transgender people, and people with substance use disorders [9]. The IDEAS method involves conducting narrative interviews with populations who have been harmed by provider stigma and creating a short film depicting experiences of healthcare inequity to be played for providers. Those interviewed are then invited to join in the showing of the film and to participate in a brief conversation with providers afterward.

The current study examined the impact and implementation of an IDEAS film that was created based on interviews with TGD Veterans about their healthcare experiences. The IDEAS intervention was delivered during one primary care existing staff meeting and one mental health grand rounds to an audience of healthcare providers in these fields. Integrating IDEAS into already existing meetings was done to reach those who may not otherwise attend a training aiming to reduce stigma. The session was introduced by the Principal Investigator (PI), who was also the occupational therapist who developed the IDEAS method. The PI explained how the IDEAS film was created, and described IDEAS as an intervention that sought to illuminate personal stories from the providers’ patient population(s). The film was played virtually, after which the PI led a discussion with the providers and TGD Veterans who were invited to join.

### Purpose

The purpose of this study was to measure the feasibility and acceptability of IDEAS implementation in VA mental health and primary care, and to assess change in pre/post provider stigma. A secondary aim of this project was to explore the methodological feasibility of examining IDEAS’ impact on TGD Veterans in preparation for a larger, national study of IDEAS’ impact on Veterans. We accomplished this secondary aim by conducting satisfaction surveys with TGD Veterans that assessed the cultural competence and cultural humility of their providers prior to and following their providers’ engagement with the IDEAS training. We used existing survey items from the Consumer Assessment of Healthcare Providers and Systems Cultural Competence item set (CAHPS-CC) [11] and a survey created by the authors designed specifically for the TGD population. This work was conducted to inform a future, large scale, national rollout of IDEAS, powered to examine the impact of provider stigma reduction on TGD Veteran healthcare experiences.

## Methods

### Design

We used a hybrid type 2 design [12] to examine IDEAS implementation acceptability, feasibility, and effectiveness (decreased provider stigma). Provider stigma is primarily assessed via a pre/post quantitative measure. Although qualitative survey data were also collected, this is not considered a mixed- or multi-methods study. Rather, qualitative survey data, assessed with basic content analysis, were reported to supplement quantitative data and serve as an illustration of provider feedback on the IDEAS experience.

We also examined the feasibility of a nested data collection methodology. We sought to explore whether collecting TGD patient perspectives on healthcare experiences surrounding IDEAS implementation could be meaningfully linked to data on provider stigma reduction resulting from IDEAS. If so, this design could be used in future studies powered to detect the impact(s) of IDEAS not only on provider stigma but also on TGD patient experiences with those providers. In addition to reporting on the feasibility of this nested design, in the current study we also report preliminary findings from this data collection with TGD Veterans.

### Participants

This study was reviewed by the Indiana University institutional review board and given exempt status – study number 16,200. Participants included (1) primary care providers and mental health providers from a Midwestern VA Medical Center who attended the IDEAS training; and (2) TGD Veterans within the Midwestern state where IDEAS was offered who had a primary care or mental health appointment within six months prior to and following IDEAS implementation with providers. All participants underwent the informed consent process.

### Measures and data collection

#### Provider data collection

We collected data from primary care and mental health providers because these two services were highly utilized by the TGD Veteran population [13, 14] Acceptability is defined as “the perception among implementation stakeholders that a given treatment, service, practice, or innovation is agreeable, palatable, or satisfactory,” and feasibility is “the extent to which a new treatment, or an innovation, can be successfully used or carried out within a given agency or setting” [15]. To measure acceptability and feasibility of IDEAS, we administered the Acceptability of Intervention Measure (AIM) and the Feasibility of Intervention Measure [15] to providers immediately after the IDEAS training. The AIM and the FIM demonstrate good substantive and discriminant content validity, structural validity, scale reliability, test-retest reliability, and sensitivity to change [16]. Questions are rated on a 5-point Likert scale from strongly disagree to strongly agree. We defined implementation success as an average score of at least 4 out of 5 (‘agree’) on both measures. Table 1 depicts the questions that comprise these measures. In addition to these quantitative measures, we included an open-ended question asking providers to share responses to the question: “Please tell us how this training impacted you.”

**Table 1:** Post-IDEAS measures of acceptability and feasibility

Acceptability of Intervention Measure (AIM)	Feasibility of Intervention Measure (FIM)
a.) IDEAS meets my approval.	a.) IDEAS seems implementable as an ongoing training.
b.) IDEAS is appealing to me.	b.) IDEAS seems possible to implement in the future.
c.) I like IDEAS.	c.) IDEAS seems doable as an ongoing training.
d.) I welcome IDEAS.	d.) IDEAS seems easy to use.

To measure change in provider stigma we administered a pre/post Acceptance and Action Questionnaire – Stigma [17]. The AAQ-S has been shown to be a valid and reliable measure of enacted stigma, with good internal consistency (Cronbach’s alpha = 0.84).^18^ The pre-survey collected demographic information alongside the AAQ-S questions and was administered as a virtual survey just prior to the IDEAS training. The post-AAQ-S questions were combined with AIM and FIM questions and the open-ended question on how they were impacted by IDEAS. Post-surveys were administered virtually immediately following the IDEAS training. Surveys prompted participants to generate a code that allowed us to examine the pre/post change in survey score for each provider while maintaining participant anonymity.

A sample size of 20 participants who completed both pre and post AAQ-S surveys achieved 90% power to detect a mean difference in AAQ-S scores between 7 and 10 points assuming a standard deviation of the differences in AAQ-S scores in the range of 7–9 points, alpha = 0.05, using a two-sided paired t-test. To account for dropout, we set our target recruitment goal at 26 providers with completed pre and post surveys. Power analysis was performed using PASS 2019.

#### TGD veteran data collection

TGD Veteran patients were identified using ICD-10 codes for gender identity disorder [18]. Self-Identified Gender Identity (SIGI) data were observed in Veteran medical records obtained via ICD-10 codes in some cases, providing further verification of TGD identities; however, SIGI data were not used as a primary means of identifying potential participants. TGD Veterans were sent a letter describing the study and providing the opportunity to opt out via a phone call to the study team and then received a follow-up call (unless they opted out) from the research assistant who shared information about study participation, answered questions, and conducted the informed consent process. Veterans who consented to participate and had a VA healthcare appointment within 6 months of IDEAS implementation engaged in a survey with the research assistant immediately following their pre-intervention mental health or primary care appointment. Pre-surveys were completed with TGD Veterans prior to their provider’s opportunity to attend IDEAS, and post-surveys were completed after their provider’s opportunity to attend IDEAS. However, to obtain as many surveys as possible, we did not exclude Veterans whose providers did not attend IDEAS. Rather, we examined our Veteran data collection alongside provider attendance at IDEAS to examine the feasibility of a nested data collection design for future study, as described below. Any TGD Veteran with primary care or mental health appointments in the recruitment window were considered eligible, and this inclusion criterion was confirmed during the initial conversation with the Veteran.

We obtained preliminary data on TGD Veteran satisfaction data using two surveys: (1) the Consumer Assessment of Healthcare Providers and Systems Cultural Competence Item Set (CAHPS-CC) [11]; and (2) the Occupational Therapy Gender Affirming Care (OGA) survey, which is an author-created survey that asks questions about aspects of healthcare that TGD patients deemed to be important. The CAHPS-CC is an open access assessment available through the Agency for Health Care Research and Quality (AHRQ) that explores several domains of “cultural competence” such as positive and negative provider communication behaviors, shared decision making, equitable treatment, and trust [11]. The OGA psychometric characteristics have yet to be studied. It was therefore solely used as an adjunct to the existing, validated CAHPS-CC items to collect information more closely tied to the TGD population and as a means of further exploration of the utility of this new instrument.

Questions for both surveys are listed in Supplementary Table 2. Questions were answered using Likert-type rating scales. Patients were asked to complete pre- and post-survey questions one time during the study period. Surveys were administered face-to-face or by phone call, and the interviewer wrote memos to capture comments and/or explanations related to responses offered by Veterans as they completed surveys.

We aimed to recruit 25% of TGD patients receiving primary care and/or mental health VA services within the study site location (one Midwestern US state) during the recruitment period, which was defined as patients with an appointment within 6 months prior to and following IDEAS implementation with providers. We deemed this percentage to be a feasible goal given the study duration and existing number of TGD patients in the healthcare system from which we were recruiting [19]. According to the local VA LGBTQ + coordinator, approximately 75 gender diverse Veterans were seeing primary care or mental health providers once or more within 6–9 months within our study setting and timeline. Thus, our feasibility benchmark was set at 18 Veterans (approximately 25% of 75). Our goal regarding feasibility was that 18 TGD Veterans would complete the informed consent process and both baseline and follow-up surveys, including the CAHPS-CC survey described above as well as the OGA. This benchmark was created as a precedent for a future national study powered to detect meaningful pre/post changes in TGD Veteran experiences with their providers. Based on the number of TGD Veterans receiving VA care nationally, a 25% percent recruitment rate would adequately facilitate this goal.

### Analyses

#### Provider stigma

AAQ-S surveys provide a composite score, with lower scores indicating reduced enacted stigma. An unadjusted two sample paired t-test was used to assess the change in AAQ-S scores in pre-post surveys. All analyses were performed in SAS 9.4 and all tests were performed using a significance level 0.05. We performed basic content analysis [20] of the qualitative feedback collected from providers via the open-ended survey question regarding their experience of IDEAS. Responses were populated in an excel sheet (Supplementary Table 1) and the study team independently rated each comment as being of positive or negative sentiment regarding IDEAS. Positive and negative responses were counted and are reported below.

#### TGD veteran surveys

This study was not powered to detect meaningful change in pre/post Veteran patient outcomes but rather, to explore the feasibility of this nested data collection method to inform a future, larger trial powered to assess the impact of IDEAS (and resulting provider stigma reduction) on TGD Veterans. We tracked how many total TGD Veterans completed both pre and post surveys.

Although pre/post change was not expected nor was it a primary aim of this study, trends in pre/post CAHPS-CC [11] and OGA surveys are reported below. These were determined by calculating the average pre and post test scores for each survey item and subtracting the pretest score from the post-test score to illustrate trends in score changes. Future studies designed to measure pre/post change in TGD Veteran experiences will employ the appropriate statistical measures to detect meaningful change.

## Results

### Provider participant characteristics

Provider participant demographic characteristics and comparison of baseline AAQ-S scores are listed in Table 2. 167 mental health providers and 52 primary care providers were invited to participate. Of those invited, 92 mental health providers and 26 primary care providers attended IDEAS. Of those who attended IDEAS, 32 mental health providers and 10 primary care providers completed both a pre and post-test. Thirty providers (both mental health and primary care) completed only a pre-test. We compared provider demographics between these two groups (those who completed only the pretest and those who completed a pre and post-test) and found no significant differences (Table 2). However, the group who completed both pre and posttests scored higher on the pretest (indicating higher stigmatizing thoughts) than those who only completed a pretest.

**Table 2:** Provider participant characteristics

Baseline Characteristic	Completed Pre and Post Test			
	No (*N* = 30)	Yes (*N* = 45)	Total (*N* = 75)	*P*-value
Age (years), n (%)				0.3684^1^
25–34	4 (13.3%)	11 (24.4%)	15 (20.0%)	
35–44	9 (30.0%)	18 (40.0%)	27 (36.0%)	
45–54	7 (23.3%)	9 (20.0%)	16 (21.3%)	
55–64	9 (30.0%)	6 (13.3%)	15 (20.0%)	
65–74	1 (3.3%)	1 (2.2%)	2 (2.7%)	
Hispanic/Latino/Spanish Origin, n (%)				0.2176^1^
No	29 (96.7%)	45 (100.0%)	74 (98.7%)	
Yes	1 (3.3%)	0 (0.0%)	1 (1.3%)	
Race, n (%)				
Black/African American, n (%)	5 (16.7%)	4 (8.9%)	9 (12.0%)	0.3099^1^
White	21 (70.0%)	35 (77.8%)	56 (74.7%)	0.4480^1^
American Indian/Alaskan Native, n (%)	0 (0.0%)	1 (2.2%)	1 (1.3%)	0.4111^1^
Asian, n (%)	1 (3.3%)	5 (11.1%)	6 (8.0%)	0.2239^1^
Native Hawaiian or Pacific Islander, n (%)	1 (3.3%)	0 (0.0%)	1 (1.3%)	0.2176^1^
Military status, n (%)				0.4191^1^
Not a service Member	28 (96.6%)	40 (88.9%)	68 (91.9%)	
Current Member	0 (0.0%)	2 (4.4%)	2 (2.7%)	
Former Member	1 (3.4%)	3 (6.7%)	4 (5.4%)	
Missing	1	0	1	
Role, n (%)				0.4927^1^
Mental health provider	18 (60.0%)	32 (72.7%)	50 (67.6%)	
Primary Healthcare Provider	11 (36.7%)	10 (22.7%)	21 (28.4%)	
Veteran Panelist	0 (0.0%)	1 (2.3%)	1 (1.4%)	
Other	1 (3.3%)	1 (2.3%)	2 (2.7%)	
Missing	0	1	1	
Highest education completed, n (%)				0.6584^1^
Associate Degree	0 (0.0%)	1 (2.2%)	1 (1.3%)	
Bachelors Degree	2 (6.7%)	1 (2.2%)	3 (4.0%)	
Masters Degree	15 (50.0%)	22 (48.9%)	37 (49.3%)	
Doctorate/Professional Degree	13 (43.3%)	21 (46.7%)	34 (45.3%)	
Gender, n (%)				0.1931^1^
Cisgender female	26 (86.7%)	31 (68.9%)	57 (76.0%)	
Cisgender male	3 (10.0%)	12 (26.7%)	15 (20.0%)	
Transgender male	0 (0.0%)	1 (2.2%)	1 (1.3%)	
Non-binary	1 (3.3%)	0 (0.0%)	1 (1.3%)	
Other	0 (0.0%)	1 (2.2%)	1 (1.3%)	

^1^Chi-Square *p*-value

### Provider stigma

Table 3 illustrates change in provider stigma. Among the 45 providers with both AAQ-S pre and post scores we found an average change score of −4.5 (11.61) among mental health providers and − 6.7 (13.85) among primary care providers, with a total average change score of −5.0 (12.04) indicating a decrease in provider stigma in both groups after engaging in IDEAS (*p* < 0.01). Change scores within this population ranged from − 54 to 10. The change scores among mental health versus primary care providers were not significantly different from one another (*p* = 0.87). No imputations were made for missing data.

**Table 3:** Pre/post change in AAQ-S

	Mental health provider	Primary Care provider	Total
N	32	10	42
Mean (SD)	−4.5 (11.61)	−6.7 (13.85)	−5 (12.04)
Median	−2.5	−1	−2
Range	−54.0, 10.0	−41.0, 8.0	−54.0, 10.0

AAQ-S score can range from 21 (low enacted stigma) to 147 (high enacted stigma)

### Feasibility, acceptability, and qualitative feedback

Regarding IDEAS acceptability, all items on the AIM were rated above 4 (agree), with a total acceptability average score of 4.33 out of 5. Average scores were as follows, with the minimum and maximum values for each item being 3 (neither agree nor disagree) and 5 (strongly agree) respectively: IDEAS meets my approval (4.39); IDEAS is appealing to me (4.34); I like IDEAS (4.32); and I welcome IDEAS (4.24). IDEAS was also feasible according to provider ratings, with a total feasibility score of 4.26 and item scores as follows: IDEAS seems implementable as an ongoing training (4.24); IDEAS seems possible to implement in the future (4.29); IDEAS seems doable as an ongoing training (4.27); and IDEAS seems easy to use (4.22). Feasibility items also had a minimum and maximum value of 3 and 5, respectively.

Twenty-four providers offered qualitative feedback about IDEAS on their post-test. All feedback was positive and spoke to the value of the film and guest speakers (e.g., “It really put a face to the issue and made me think about what I can do to ensure that I am treating trans Veterans with the care and respect they deserve”) as well as the impact IDEAS had on providers’ motivation and self-efficacy to initiate change. For instance, one provider noted: “The first vignette in the video posed a challenge to providers to help shift the culture of the VA…this is a lofty but important goal that I would love to participate in.” Another expressed that: “Some of it was really eye opening and makes me feel more sensitive to the good and bad experiences that might emerge. I feel more empowered that my interactions with transgender folks can make a big difference.” Qualitative remarks are listed in Supplementary Table 1.

### Impact of IDEAS on gender diverse veterans

A search using ICD-10 codes yielded 141 TGD Veterans. Of those, we were able to reach forty-two by phone. One reported they were not receiving any VA care, and 4 declined participating. The remaining 37 completed a presurvey. Twenty-one TGD Veterans who completed a pre-survey had a provider appointment scheduled within 6 months following IDEAS, and of those 21, 18 completed post surveys and 3 declined. We therefore met our goal of obtaining both a pre and post survey from at least 18 TGD Veterans. Post-survey completion was limited by project staffing changes which posed challenges to follow-up data collection.

Supplementary Table 2 describes TGD Veteran participant characteristics as well as average pre/post scores on both surveys (CAHPS-CC and Occupational Therapy Gender Affirming Care Survey) and the difference between pre and post averages per item and total. Although this study was not powered to detect meaningful pre/post change on TGD Veteran surveys, it is worth noting the trend in pre/post scores on both surveys. All but one of the CAHPS-CC items improved from baseline to follow-up, suggesting a positive trend in which Veterans perceived providers to be demonstrating increased cultural competence after IDEAS training. No item scores decreased from baseline to follow-up, and one item remained the same: *In the last 12 months*,* how often have you been treated unfairly at this doctor’s office because of the type of health insurance you have or because you don’t have health insurance?* The average response to this item at baseline and follow-up was 4 (Never), which makes sense given that private health insurance status does not influence access to VA healthcare. On the Occupational Therapy Gender Affirming Care Survey only one item decreased from baseline to follow-up: *My provider explains the purpose of medical procedures and professionals who are involved in my care*. The average baseline score of 4.68 (agree/strongly agree) decreased by 0.18 points to 4.50, still falling in the agree/strongly agree range.

### Qualitative observations during veteran interviews

The research assistant took memos while conducting pre and post surveys with TGD Veterans. Some observations from these memos illustrate factors that may have impacted scores and could provide directions for future evaluation. For example, one autistic non-binary young person scored their provider notably lower than average on the pre-CAHPS-CC, reporting that they felt negatively about most healthcare experiences, due to providers not allowing time for cognitive processing.

Second, it was consistently noted that Veterans over 55 described generally lower standards for gender affirming care. For example, a positive assessment of a provider may be one that does not blatantly deny the existence of gender diversity, even if the provider was not knowledgeable regarding the range of gender diverse identities and associated specific care considerations. Veterans over 55 were more likely to describe having a long relationship with their provider, whom they felt knew them as a person, which they reported contributed to positive perceptions of providers even when the providers were not knowledgeable about their gender and related healthcare needs. By contrast, younger TGD Veterans had higher expectations that providers were knowledgeable and affirming about their identities and care requirements.

Third, several respondents noted that one of the survey questions regarding whether providers spend enough time with the patient is perceived as an issue with the VA system rather than the provider and therefore Veterans were resistant to score providers low on this item.

Fourth, Veterans noted that care from residents was markedly less affirming than care from their typical attending providers, indicating the importance of gender affirming care training requirements for trainees as they step into their professional identities and responsibilities as primary care and mental health providers.

Finally, Veterans noted that front desk staff within their healthcare system may misgender them over the phone, even when they believe that pronouns are clearly visible within the Veteran’s medical record.

### Feasibility of nested data collection design

We recorded the primary care and mental health providers of the TGD Veterans interviewed to determine the feasibility of a nested design for future study. However, most providers only had one TGD Veteran from our sample on their caseload. Each TGD Veteran interviewed had a distinct mental health provider and 15 TGD Veterans had distinct primary care providers. One physician was the primary care provider for 7 TGD Veteran participants; another was the provider for 4 TGD Veteran participants; two were providers for 3 TGD Veteran participants, and 3 were providers for 2 TGD Veteran participants. Due to the small number of TGD Veteran participants seen by a common provider, we determined the nested design to have limited feasibility in the current study. Future studies may benefit from the seeking out of providers with large TGD caseloads to increase the feasibility of a nested design for testing the impact of IDEAS provider trainings on TGD Veterans.

## Discussion

### Change in provider stigma

We observed an average pre/post change score of −5 among healthcare providers, indicating the possibility that IDEAS may decrease enacted provider stigma. The AAQ-S measures psychological flexibility and inflexibility regarding stigmatizing thoughts, with decreasing enacted stigma being conceptualized as an increase in the former and a decrease in the latter. An improved score suggests a provider may still experience stigmatizing thoughts but has greater flexibility regarding whether and how they respond to such thoughts [17]. Future research that explores qualitative feedback from providers over time may be useful in determining if and how IDEAS impacts provider interactions with TGD patients and may add insight to these quantitative findings. Our preliminary content analysis of qualitative survey feedback suggests providers experienced IDEAS as a positive opportunity for growth. However, future ongoing research is needed to explore provider experiences of IDEAS and its impact over time as it relates to patient care.

### TGD veteran perspectives

As noted above, TGD survey scores may at times be more reflective individual patient factors rather than provider behaviors, as noted by the TGD Veteran with autism who noted rating providers low across the board. This is reflective of existing literature documenting that gender diverse patients with comorbid conditions face greater health disparities, resulting in lower overall patient satisfaction [21]. TGD Veterans also expressed that some negative healthcare experiences are due to the actions of administrative staff or larger systemic issues. A study by Kcomt [22] discussed the importance of addressing institutional policies to decrease profound discrimination of transgender and gender diverse patients. Our findings reflect the importance of evaluating the larger institutional factors impacting healthcare quality, which would not be captured by surveys about satisfaction with healthcare providers.

### Limitations and future research

This study’s findings are limited to VA healthcare facilities within one Midwestern state and primarily referred to experiences within a large VA hospital in a central, urban location. As such, Veteran survey responses do not adequately reflect TGD VA experiences across the United States. For example, one Veteran mentioned traveling across state lines to receive gender affirming care. This Veteran expressed that their survey responses were specific to the study location (which is where they traveled to for care) but would misrepresent the other VA system in which they had received less gender-affirming care. They specifically discussed their perception that the VA was not as a whole gender affirming, requiring them to seek out specific places within the VA that were equipped to provide them with good care. Given this perception of the VA system in which our study was set, findings (largely positive patient satisfaction pre and post IDEAS) should be interpreted in this context. The contrast in quality of TGD healthcare based on location has been echoed by Gonzales [23] which found TGD adults who seek care in states with stronger TGD protections report less mental distress versus their TGD peers who seek care in states with fewer TGD protections.

While systemic and structural issues have been shown to negatively impact TGD healthcare experiences [22], IDEAS is limited to addressing the harms caused directly by providers. The measures used in this study may reflect healthcare experiences related to factors beyond interactions with providers. For example, negative satisfaction surveys could larger problems within healthcare systems rather than problems with providers themselves, highlighting a limitation of this work. Future examination of these nuances and efforts to mores specifically assess provider interactions may be helpful in addressing this limitation.

This study is further limited in that TGD Veteran data collection was performed by a single interviewer; thus, memos are derived from only one researcher’s observations which were then interpreted by the research team of cisgender, white women. Further, it is possible that implicit biases of the research team could contribute to analysis bias and/or confirmation bias, which has been described as occurring when research questions or methods influence the ways in which participants respond so that they come to match the researcher’s expectations [24]. In addition, the exclusively positive remarks about IDEAS from providers and responses in general from TGD Veterans may be a reflection of positive response bias, in which individuals provide responses that are favorable and socially acceptable rather than providing feedback on how they truly feel [25]. 

Future research is needed to further explore the impact of IDEAS on providers and TGD Veterans. A secondary aim of this study was to pilot the feasibility of a nested data collection design. However, most TGD Veterans who were interviewed did not share providers, limiting the feasibility of this design. In future work it may be useful to identify and over-recruit providers with large numbers of TGD Veteran patients to engage in IDEAS. This would facilitate a nested design in which we could examine the impact of IDEAS on those providers and the TGD Veterans they serve. However, providers with large TGD caseloads may possess confounding traits that may limit generalizable to a larger provider population. In the current study we did not perform pre/post statistical tests on Veteran data and therefore also did not examine potential confounders such as the number of appointments Veterans had with a provider prior to the study, which could likely influence the relationship a Veteran had with a provider. Such variables will be important to consider in future work.

Another limitation is that IDEAS implementation in the future may be impeded by executive orders reducing recognition of and programming for the TGD population. However, many educational curricula accreditation standards maintain emphasis on training future healthcare professionals in how to serve diverse populations. Thus, IDEAS implementation may compliment educational programs for healthcare professional students and medical residents with Veteran patient caseloads.

Finally, IDEAS was designed by occupational therapists to increase occupational justice in the realm of healthcare for patients who have been harmed by provider bias across disciplines. IDEAS may be improved by consulting a wide range of healthcare providers and incorporating their perspectives and insights into the IDEAS trainings.

### Improving care for TGD veterans

Studies show that provider stigma is associated with lower healthcare utilization among TGD Veterans [2, 26] which, in turn, can increase adverse health outcomes for this population [4]. As such, alleviating provider stigma through interventions such as IDEAS has the potential to improve health outcomes for TGD Veteran patients. This is in line with prior studies suggesting that reduced provider stigma and increasing high-quality communication with providers buffers negative health behaviors and outcomes among LGBT Veterans [27]. Furthermore, reducing provider stigma to potentially increase healthcare utilization among TGD Veterans may support several VA priorities such as decreasing suicide risk and increasing mental health among Veterans, both of which disproportionally affect TGD Veterans [3, 4]. 

## Conclusions

IDEAS is a feasible, acceptable, and potentially effective intervention for reducing provider biases that may negatively impact healthcare equity for TGD Veterans. Further research is needed to measure the impact of IDEAS provider trainings on the care of TGD Veteran patients and the feasibility of nested data collection to show this impact. However, preliminary results are promising regarding the perceptions of providers following IDEAS trainings among TGD Veterans. Existing open-access surveys such as the CAHPS-CC items capture some information regarding healthcare experiences but may benefit from additional items that inquire about priorities identified by TGD patients. Further evaluation of such assessments is warranted.

## Supplementary Information

Supplementary Material 1

Supplementary Material 2

## Data Availability

Data are provided within the manuscript - supplementary information is available by emailing the corresponding author.

## Declarations

### Disclaimer

This work describes the views of the authors and not the US Government.

## Abbreviations

VA: Veterans Affairs
TGD: Transgender and gender diverse
AIM: Acceptability of Intervention Measure
FIM: Feasibility of Intervention Measure
AAQ-S: Acceptance and Action Questionnaire – Stigma
CAHPS-CC: Consumer Assessment of Healthcare Providers and Systems – Cultural Competence
OGA: Occupational Therapy Gender Affirming Care
ICD: International Classification of Diseases
AHRQ: Agency for Healthcare Research and Quality

## References

[1] Gates GJ, Herman JL. Transgender military service in the United States. The Williams Institute at UCLA School of Law. 2014. Available from: http://www.jstor.org/stable/resrep35579.

[2] Schvey NA, Klein DA, Pearlman AT, Kraff RI, Riggs DS. Stigma, health, and psychosocial functioning among transgender active duty service members in the US military. Stigma Health. 2020;5(2):188. 10.1089/trgh.2019.0044 PMC7906232 33644309

[3] Boyer TL, Youk AO, Haas AP, Brown GR, Shipherd JC, Kauth MR, et al. Suicide, homicide, and all-cause mortality among transgender and cisgender patients in the veterans health administration. LGBT Health. 2021;8(3):173–80. 10.1089/lgbt.2020.0235. 33544021 10.1089/lgbt.2020.0235

[4] Brown GR, Jones KT. Mental health and medical health disparities in 5135 transgender veterans receiving healthcare in the veterans health administration: a case–control study. LGBT Health. 2015;3(2):122–31. 10.1089/lgbt.2015.0058. 26674598 10.1089/lgbt.2015.0058

[5] Correro AN, Hinrichs KL, Nathan S. My life, my story and identity disclosure among transgender and gender diverse veterans: A program evaluation. Transgender Health. 2021;7(6):556–60. 10.1089/trgh.2021.0004. 10.1089/trgh.2021.0004 PMC9829157 36644119

[6] Blosnich JR, Marsiglio MC, Gao S, Gordon AJ, Shipherd JC, Kauth M, et al. Mental health of transgender veterans in US states with and without discrimination and hate crime legal protection. Am J Public Health. 2016;106(3):534–40. 10.2105/ajph.2015.302981. 26794162 10.2105/AJPH.2015.302981 PMC4815748

[7] Kauth MR, Blosnich JR, Marra J, Keig Z, Shipherd JC. Transgender health care in the US military and veterans health administration facilities. Curr Sex Health Rep. 2017;9:121–7.

[8] Shipherd JC, Kauth MR, Firek AF, Garcia R, Mejia S, Laski S, et al. Interdisciplinary transgender veteran care: development of a core curriculum for VHA providers. Transgend Health. 2016;1(1):54–62. 10.1089/trgh.2015.0004. 29159298 10.1089/trgh.2015.0004 PMC5685249

[9] Wasmuth S, Belkiewitz J, Miech E, Li C, Harris A, Hernandez J, et al. A hybrid type III analysis of a filmed story-telling intervention’s impact of provider stigma. OTJR Occup Ther J Res. 2024;1–10. 10.1177/15394492241260022. 10.1177/15394492241260022 PMC13290392 39086138

[10] Wasmuth S, Leonhardt B, Pritchard K, Li CY, DeRolf A, Mahaffey L. Supporting occupational justice for transgender and gender-nonconforming people through narrative-informed theater: a mixed-methods feasibility study. Am J Occup Ther. 2021;75(4):1–12. 10.5014/ajot.2021.045161. 10.5014/ajot.2021.045161 PMC8369667 34780605

[11] Weech-Maldonado R, Carle A, Weidmer B, Hurtado M, Ngo-Metzger Q, Hays RD. The consumer assessment of healthcare providers and systems (CAHPS) cultural competence (CC) item set. Med Care. 2012;50:S22–31. 10.1097/MLR.0b013e318263134b. 22895226 10.1097/MLR.0b013e318263134b PMC3748811

[12] Kemp CG, Wagenaar BH, Haroz EE. Expanding hybrid studies for implementation research: intervention, implementation strategy, and context. Front Public Health. 2019;7:325. 10.3389/fpubh.2019.00325. 31781528 10.3389/fpubh.2019.00325 PMC6857476

[13] Lee JL, Hirsh A, Radhakrishnan A, Jasuja GK, Taylor S, Dickinson S, et al. Mental health utilization among transgender veterans. JAMA Netw Open. 2025;8(1):e2454694. 10.1001/jamanetworkopen.2024.54694. 39804641 10.1001/jamanetworkopen.2024.54694 PMC11731220

[14] Copeland LA, Wolfe HL, Jackson SS, Buljubasic N, Kauth MR, Hashemi L. Treating transgender and gender-diverse veterans in the veterans health administration: 23 years of findings. LGBT Health. 2025. 10.1089/lgbt.2024.0314. 39964769 10.1089/lgbt.2024.0314 PMC12369840

[15] Proctor E, Silmere H, Raghavan R, Hovmand P, Aarons G, Bunger A, et al. Outcomes for implementation research: conceptual distinctions, measurement challenges, and research agenda. Adm Policy Ment Health. 2010;38(2):65–76. 10.1007/s10488-010-0319-7. 10.1007/s10488-010-0319-7 PMC3068522 20957426

[16] Weiner BJ, Lewis CC, Stanick C, Powell BJ, Dorsey CN, Clary AS, et al. Psychometric assessment of three newly developed implementation outcome measures. Implement Sci. 2017;12(1): 108. 10.1186/s13012-017-0635-3. 28851459 10.1186/s13012-017-0635-3 PMC5576104

[17] Levin ME, Luoma JB, Lillis J, Hayes SC, Vilardaga R. The acceptance and action Questionnaire – Stigma (AAQ-S): developing a measure of psychological flexibility with stigmatizing thoughts. J Contextual Behav Sci. 2013;3(1):21–6. 10.1016/j.jcbs.2013.11.003. 10.1016/j.jcbs.2013.11.003 PMC4255270 25485230

[18] Hill LW, Reisman JI, Yoon SS, et al. Validating Data-Driven methods for identifying transgender individuals in the veterans health administration of the US department of veterans affairs. Am J Epidemiol. 2021;190(9):1928–34. 10.1093/aje/kwab102. 34467408 10.1093/aje/kwab102

[19] Stewart AL, Nápoles AM, Piawah S, Santoyo-Olsson J, Teresi JA. Guidelines for evaluating the feasibility of recruitment in pilot studies of diverse populations: an overlooked but important component. Ethn Dis. 2020;30(Suppl):745–54. 10.18865/ed.30.s2.745. 33250621 10.18865/ed.30.S2.745 PMC7683033

[20] Drisko J, Maschi T. Content analysis. 2015. 10.1093/acprof:oso/9780190215491.001.0001.

[21] Wolfe HL, Vimalananda VG, Wong DH, Reisman JI, Rao SR, Shipherd JC, et al. Patient characteristics associated with receiving gender-affirming hormone therapy in the veterans health administration. Transgend Health. 2022;9(2):151–61. 10.1089/trgh.2022.0040. 10.1089/trgh.2022.0040 PMC11059777 38694620

[22] Kcomt L. Profound health-care discrimination experienced by transgender people: rapid systematic review. Soc Work Health Care. 2018;58(2):201–19. 10.1080/00981389.2018.1532941. 30321122 10.1080/00981389.2018.1532941

[23] Gonzales G, Tran NM, Bennett MA. State policies and health disparities between transgender and cisgender adults: considerations and challenges using population-based survey data. J Health Polit Policy Law. 2022;47(5):555–81. 10.1215/03616878-9978117. 35576319 10.1215/03616878-9978117

[24] Peters U. What is the function of confirmation bias? Erkenntnis. 2022;87(3):1351–76.

[25] Elston D. Participation bias, self-selection bias, and response bias. J Am Acad Dermatol. 2021. 10.1016/j.jaad.2021.06.025. Accessed 12 June 2024. 10.1016/j.jaad.2021.06.025 34153389

[26] Johnson N, Pearlman AT, Klein DA, Riggs D, Schvey NA. Stigma and barriers in health care among a sample of transgender and gender-diverse active duty service members. Med Care. 2023;61(3):145–9. 36728493 10.1097/MLR.0000000000001818

[27] Ruben MA, Livingston NA, Berke DS, Matza AR, Shipherd JC. Lesbian, gay, bisexual, and transgender veterans’ experiences of discrimination in health care and their relation to health outcomes: a pilot study examining the moderating role of provider communication. Health Equity. 2019;3(1):480–8. 31559377 10.1089/heq.2019.0069 PMC6761590

