# Supplementary material for: A pre/post study of a narrative “IDEAS” intervention’s impact on provider stigma and feasibility of collecting transgender and gender diverse veteran care experiences
Source: BMC Public Health. 2025 Oct 1;25:3280. doi: 10.1186/s12889-025-24494-2 (PMC12486660; doi:10.1186/s12889-025-24494-2)
Supplement: Supplementary file 1 — Supplementary Material 1 [file 12889_2025_24494_MOESM1_ESM.docx]

Supplementary Table 1. Provider Open-Ended Feedback Post-IDEAS

| 1 | Good hearing about our customers’ experiences |
| --- | --- |
| 2 | Having Veteran panelists was very helpful |
| 3 | Very informative and great to hear from those actually affected and going through some of these experiences |
| 4 | Was helpful |
| 5 | Good training |
| 6 | An eye-opener and very helpful! |
| 7 | Great info and time for reflection |
| 8 | This was excellent! The panelists were amazing and should be validated by all providers every day |
| 9 | I enjoyed listening to the guests who talked about their experiences as trans individuals. I hope I can help chip away at the stigma surrounding this issue in the VA |
| 10 | Just sharing my appreciation for the panelists and their willingness to provide feedback to us as providers |
| 11 | I appreciated hearing feedback from the trans Veterans which assists myself in being more aware to their potential experiences |
| 12 | This training was excellent and very useful. The first vignette in the video posed a challenge to providers to help shift the culture of the VA so that the Vets themselves are more affirming. I hope this is an ongoing conversation as this is a lofty yet IMPORTANT goal that I would love to participate in. Excellent presentation. Highly recommend to others! |
| 13 | I appreciated the panel approach that allowed individuals within the LGBTQIA+ community to share their experiences without making it their responsibility to educate |
| 14 | It was thought provoking. It really put a face to the issue and made me think about what I can do to ensure that I am treating trans Veterans with the care and respect they deserve. Very informative. I greatly appreciate the panel’s input. |
| 15 | I loved hearing the thoughts and experiences from transgender folks. Some of it was really eye opening and makes me feel more sensitive to the good and bad experiences that might emerge. I feel more empowered that my interactions with transgender folks can make a big difference |
| 16 | I appreciated the realness of the stories and how applicable they are to our work at the VA |
| 17 | Great presentation! |
| 18 | Very helpful/interesting |
| 19 | Thanks! |
| 20 | Great experience |
| 21 | It was great, thank you! |
| 22 | Loved the panelist portion |
| 23 | I really appreciate the willingness of the Veterans to speak about their experiences at the VA!  I liked the conversational/panel format of the meeting.  I would like to learn more about ways we can help keep LGBTQ+ Veterans safe outside of our offices (waiting rooms, etc). |
| 24 | Really enjoyed the video and hearing directly from Veterans about specifics |
