# Supplementary material for: A pre/post study of a narrative “IDEAS” intervention’s impact on provider stigma and feasibility of collecting transgender and gender diverse veteran care experiences
Source: BMC Public Health. 2025 Oct 1;25:3280. doi: 10.1186/s12889-025-24494-2 (PMC12486660; doi:10.1186/s12889-025-24494-2)
Supplement: Supplementary file 2 — Supplementary Material 2 [file 12889_2025_24494_MOESM2_ESM.docx]

Supplementary Table 2. TGD Veteran Characteristics and Survey Responses

| **Demographic Characteristics** | **Frequency** | **Percentage** |  |
| --- | --- | --- | --- |
| Age |  |  |  |
| 18 - 34 | 6 | 16 |  |
| 35 - 64 | 20 | 54 |  |
| 65+ | 10 | 27 |  |
| No answer | 1 | 3 |  |
| Ethnicity |  |  |  |
| Hispanic, Latino, or Spanish origin? | 0 | 0 |  |
| Race* | | | |
| White | 34 | 92 |  |
| Black or African American | 4 | 11 |  |
| American Indian or Alaska Native | 3 | 8 |  |
| Military Status | | | |
| Current service member | 2 | 5 |  |
| Former service member | 34 | 92 |  |
| No answer | 1 | 3 |  |
| Education | | | |
| Less than high school diploma | 1 | 3 |  |
| High school degree or equivalent | 6 | 16 |  |
| Some college, no degree | 8 | 22 |  |
| Associate degree | 8 | 22 |  |
| Bachelor’s degree | 8 | 22 |  |
| Master’s degree | 4 | 11 |  |
| Doctorate or professional degree | 1 | 3 |  |
| No answer | 1 | 3 |  |
| Gender | | | |
| Transgender female | 18 | 49 |  |
| Transgender male | 11 | 30 |  |
| Non-binary | 3 | 8 |  |
| Other | 4 | 11 |  |
| No answer | 1 | 3 |  |
| **note.* Numbers for race do not equal 37 or 100% due to identification with multiple races among participants | | | |
| **CAHPS-CC Survey** | **Pre (max/min)** | **Post (max/min)** | **Change** |
| Communication – Positive Behaviors (1 Never, 2 Sometimes, 3 Usually, 4 Always) | | | |
| *In the last 12 months, how often did this doctor explain things in a way that was easy to understand?* | 3.65(4/2) | 3.76(4/3) | 0.11 |
| *In the last 12 months, how often did this doctor listen carefully to you?* | 3.68(4/2) | 3.88(4/3) | 0.20 |
| *In the last 12 months, how often did this doctor spend enough time with you?* | 3.49(4/2) | 3.76(4/2) | 0.27 |
| *In the last 12 months, how often did this doctor show respect for what you had to say?* | 3.92(4/3) | 4.0(4/4) | 0.08 |
| *In the last 12 months, how often did this doctor give you easy to understand instructions about taking care of these health problems or concerns?* | 3.68(4/2) | 3.82(4/3) | 0.14 |
| Communication – Negative Behaviors (1 Always, 2 Usually, 3 Sometimes 4 Never) | | | |
| *In the last 12 months, how often did this doctor interrupt you when you were talking?* | 3.81(4/2) | 3.88(4/3) | 0.07 |
| *In the last 12 months, how often did this doctor talk too fast when talking with you?* | 3.73(4/2) | 3.82(4/3) | 0.09 |
| Communication – Negative Behaviors (1 Yes Definitely, 2 Yes Somewhat, 3 No) | | | |
| *In the last 12 months, did this doctor ever use a condescending, sarcastic, or rude tone or manner with you?* | 2.97(3/2) | 3.0(3/3) | 0.03 |
| Shared Decision Making (0 No, 1 Yes) | | | |
| *In the last 12 months, did this doctor talk with you about the pros and cons of each choice for your treatment or health care?* | 0.76(1/0) | 0.88(1/0) | 0.12 |
| *In the last 12 months, when there was more than one choice for your treatment or health care, did this doctor ask which choice you thought was best for you?* | 0.73(1/0) | 0.94(1/0) | 0.21 |
| Equitable Treatment (1 Always, 2 Usually, 3 Sometimes, 4 Never) | | | |
| *In the last 12 months, how often have you been treated unfairly at this doctor's office because of your race or ethnicity?* | 3.95(4/3) | 4(4/4) | 0.05 |
| *In the last 12 months, how often have you been treated unfairly at this doctor's office because of the type of health insurance you have or because you don't have health insurance?* | 4.0(4/4) | 4.0(4/4) | 0.0 |
| Trust (0 No, 1 Yes) | | | |
| *Do you feel you can tell this doctor anything, even things that you might not tell anyone else?* | 0.78(1/0) | 0.94(1/0) | 0.16 |
| *Do you trust this doctor with your medical care?* | 0.95(1/0) | 1.0(1/1) | 0.05 |
| *Do you feel this doctor always tells you the truth about your health, even if there is bad news?* | 0.97(1/0) | 1.0(1/1) | 0.03 |
| *Do you feel this doctor cares as much as you do about your health?* | 0.84(1/0) | 0.94(1/0) | 0.10 |
| Trust (1 Never, 2 Sometimes, 3 Usually, 4 Always) | | | |
| *In the last 12 months, how often did you feel this doctor really cared about you as a person?* | 3.59(4/1) | 4.0(4/4) | 0.41 |
| **Occupational Therapy Gender Affirming Care Survey**  Higher scores indicated more desirable services. All items scored as follows unless otherwise indicated by * (1 Strongly disagree, 2 Somewhat disagree, 3 Neither disagree nor agree, 4 Agree, 5 Strongly Agree) | | | |
| *My provider listens carefully to me.* | 4.68 | 5 | 0.32 |
| *When there is more than one choice for my treatment or health care, my provider asks which choice I think is best for me.* | 4.35 | 4.61 | 0.26 |
| *I feel my provider always tells me the truth about my health, even if there is bad news.* | 4.81 | 5 | 0.19 |
| *My provider explains things in a way that is easy to understand.* | 4.51 | 4.83 | 0.32 |
| *My provider shows respect for what I have to say.* | 4.70 | 4.94 | 0.24 |
| *I feel my provider spends enough time with me.* | 4.14 | 4.83 | 0.69 |
| **I feel that I am treated unfairly at my provider's office because of my race, ethnicity, or gender identity.* (Reverse scored: 1 Strongly agree, 2 Somewhat agree 3 Neither agree nor disagree, 4 Somewhat disagree, 5 Strongly disagree) | 4.70 | 4.83 | 0.13 |
| *I trust my provider with my medical care.* | 4.75 | 5 | 0.25 |
| *My provider believes me when I share my health concerns.* | 4.59 | 4.83 | 0.24 |
| *My provider is knowledgeable about my gender identity and related healthcare treatment.* | 4.27 | 4.83 | 0.56 |
| **I must advocate for myself in order for my provider to treat me with appropriate care related to my gender identity.* (Reverse scored: 1 Strongly agree, 2 Somewhat agree 3 Neither agree nor disagree, 4 Somewhat disagree, 5 Strongly disagree) | 3.46 | 4.39 | 0.93 |
| *My provider acknowledges all of my identities when providing holistic care.* | 4.43 | 4.61 | 0.18 |
| *My provider explains the purpose of medical procedures and professionals who are involved in my care.* | 4.68 | 4.50 | 0.18 |
| *All the staff at my provider's office affirm my gender.* | 3.92 | 4.22 | 0.30 |
| *My provider listens to my feedback and makes appropriate changes.* | 4.43 | 4.89 | 0.46 |
| *My provider fully addresses and resolves my medical concerns* | 4.39 | 4.50 | 0.11 |
| **My provider asks me to educate them about my gender identity, and/or I need to educate my provider to ensure that I am provided proper care.* (Reverse scored: 1 Strongly agree, 2 Somewhat agree 3 Neither agree nor disagree, 4 Somewhat disagree, 5 Strongly disagree) | 3.27 | 4.12 | 0.85 |
| *(referring to prior question) Is this a positive thing or is it a frustrating thing?* (0 Not applicable, 1 Frustrating, 2 Positive) | 0.86 | 0.86 | 0 |
| *My provider acknowledges and understands my gender identity.* | 4.54 | 4.88 | 0.34 |
| *My provider acknowledges the potential dysphoria related to certain tests and procedures and takes steps to alleviate the potential for dysphoric harm.* | 3.83 | 4.35 | 0.52 |
| *My provider is sensitive to potential dysphoria and limits requests to ask me to take off my clothes.* | 3.78 | 4.24 | 0.46 |
| **Total Score/Average Score** | **87.09(4.15)** | **94.26(4.49)** | **7.17(0.34)** |

******Green indicates improved scores from baseline to follow-up; Red indicates reduced scores from baseline to follow-up*
